# Insight on the Genetics of Atrial Fibrillation in Puerto Rican Hispanics

**DOI:** 10.1155/2021/8819896

**Published:** 2021-01-07

**Authors:** Ariel F. Gonzalez-Cordero, Jorge Duconge-Soler, Hilton Franqui-Rivera, Roberto Feliu-Maldonado, Abiel Roche-Lima, Israel Almodovar-Rivera

**Affiliations:** ^1^Department of Medicine, Cardiovascular Disease Division, University of Puerto Rico, San Juan 00936, Puerto Rico, USA; ^2^Department of Pharmaceutical Sciences, School of Pharmacy, University of Puerto Rico Medical Sciences Campus, San Juan 00936, Puerto Rico, USA; ^3^Center for Collaborative Research in Health Disparities (CCRHH), University of Puerto Rico Medical Sciences Campus, San Juan 00936, Puerto Rico, USA; ^4^Department of Biostatistics and Epidemiology, Graduate School of Public Health, San Juan 00936, Puerto Rico, USA

## Abstract

Non-Hispanic whites present with higher atrial fibrillation (AF) prevalence than other racial minorities living in the mainland USA. In two hospital-based studies, Puerto Rican Hispanics had a lower prevalence of atrial fibrillation of 2.5% than non-Hispanic Whites with 5.7%. This data is particularly controversial because Hispanics possess a higher prevalence of traditional risk factors for developing AF yet have a lower AF prevalence. This phenomenon is known as the atrial fibrillation paradox. Despite recent advancements in understanding AF, its pathogenesis remains unclear. In this study, we compared a genetic dataset of Puerto Rican Hispanics to 111 SNP known to be associated with AF in a large European cohort and determine if they are associated with AF susceptibility in our cohort. To achieve this aim, we performed a secondary analysis of existing data using the following two studies: (1) *The Pharmacogenetics of Warfarin in Puerto Ricans study* and the (2) *A Genomic Approach for Clopidogrel in Caribbean Hispanics*, and assess for the presence of European SNPs associated with AF from the genome-wide association study of 1 million people identifies 111 loci for atrial fibrillation. We used data from 555 cardiovascular Puerto Rican Hispanic patients, consisting of 486 control and 69 cases. We found that the following SNPs showed significant association with AF in PHR: rs2834618, rs6462079, rs7508, rs2040862, and rs10458660. Some of these SNPs are proteins involved in lysosomal activities responsible for breaking ceramides to sphingosines and collagen deposition around atrial cardiomyocytes. Furthermore, we performed a machine learning analysis and determined that Native American admixture and heart failure were strongly predictive of AF in PHR. For the first time, this study provides some genetic insight into AF's mechanisms in a Puerto Rican Hispanic cohort.

## 1. Introduction

Atrial fibrillation (AF) is a common arrhythmia worldwide that can cause cardioembolic events that travel to the brain and cause permanent neurological damage. AF affects more than 6 million individuals in the United States, and this number is expected to double by the year 2050 as the population ages [1]. There are risk factors that can predispose to develop atrial fibrillation. One of the most influential risk factors is older age, diabetes, hypertension, structural heart disease, sleep apnea, and excessive alcohol ingestion [2, 3]. Despite scientific advancements in this field, we do not have enough insight into AF's pathogenesis.

Multiethnic epidemiological studies have revealed that non-Hispanic Whites (NHW) have a higher prevalence of AF, almost a twofold higher incidence of AF, compared to Hispanics, Blacks, and Asians [4]. This fact may be counterintuitive, particularly among Hispanics, because of their higher prevalence of common risk factors for AF (i.e., higher rates of metabolic syndrome and diabetes) [5, 6]. It is expected that the more risk factors you have, the higher its prevalence. This phenomenon is known as the atrial fibrillation paradox. This racial paradox could suggest NHW possess specific genetics that predisposes them to AF [7]. Such is the conclusion of a genome-wide association study (GWAS) in African Americans (AA), which identified European ancestry as an independent risk factor for developing AF. Furthermore, a large multiethnic study using genome-wide association (GWAS) identified 97 genes strongly associated with AF development. These genes' relationship was homogeneous among the studied ethnicities: Brazilian, British/Irish, and Japanese individuals. Some of these genes included PITXc, SCNA5A, KCNH2, KCNJ5, TBX3-5, NKX2-5, and PRRX1 [8]. However, other studies have uncovered that the effects of AF-associated genes can vary by ethnicity. For example, the rs10824026 SNP at the 10q22 chromosome is strongly associated with AF but has shown a disproportionately higher risk of AF among NHW compared to AA [9].

Unfortunately, atrial fibrillation GWAS in racial minorities is lacking, and its ethnic-specific genetic basis unknown [10]. For this reason, this study's goal was to perform a comparative genetic analysis of Hispanics living in the Island of Puerto Rico using AF-single nucleotide polymorphisms (SNP) specific to Europeans. We used the results from a large GWAS based on 1 million Europeans, which identified approximately 111 SNPs associated with AF [11]. These reported SNPs represent the strongest genetic contenders to explain AF in Europeans at the time of this publication. This study represents the first analysis of European-specific risk factors for AF in Puerto Rican Hispanics.

## 2. Materials and Methods

### 2.1. Study Design

The following two studies were used to generate a genetic dataset for the secondary analysis of this research protocol: (1) *The Pharmacogenetics of Warfarin in Puerto Ricans study* [12] and the (2) *A Genomic Approach for Clopidogrel in Caribbean Hispanics* [13]. We used data from 555 cardiovascular PRH patients, including those who have a diagnosis of atrial fibrillation. All individual genetic samples were previously interrogated in a CLIA-certified lab (LPH, Genomas Inc., Hartford, CT) to identify relevant single nucleotide polymorphisms (SNPs) in multiple loci across the whole genome. They used the Infinium™ Human OminiXpress-24 BeadChip array (~650 K markers; warfarin study) or the Infinium™ Multi-Ethnic AFR/AMR Mega chip array (~1.4 M markers, clopidogrel study) genetic panels commercially available by the Illumina® company (San Diego, CA, USA). The corresponding VCF files of genotypes and haplotypes at the genome-wide level for each individual were generated. Such datasets were first visualized and revised for consistency and data quality controls (QC) following standard procedures. Records with significant missing values were removed from further analysis; however, some records with a few missing values were still used in the subsequent analysis if the proposed bioinformatics techniques allowed. Afterward, we made queries to retrieved the genotypes/haplotypes that matched a list of 111 relevant SNPs previously found to be associated with atrial fibrillation in Europeans [11]. The retrieved genetic information from participants was then assembled to another worksheet (excel file) containing their corresponding clinical and demographic covariates (e.g., age, weight, gender, indication, smoking status, comedications, comorbidities, ancestry proportions), which were used together in a subsequent analysis of association. To this end, a *candidate gene association analysis* was performed in our population to replicate prior findings (i.e., 111 relevant signals) from a genome-wide association study (GWAS) of atrial fibrillation in European individuals. Likewise, different machine-learning (ML) algorithms were also tested to identify optimal prediction models for atrial fibrillation risk in Puerto Ricans. All participants from the studies mentioned above kindly provided a broad consent for future data analyses as part of the corresponding IRB approval.

### 2.2. European Biobank Genomic Analysis

Nielsen et al. published a study that identified 111 candidate genes associated with AF in Europeans with *p* value <5 × 10^−8^. This genome-wide association study (GWAS) used a total of 1,030,836 European patients, where 60,620 had AF (case group) and 970,216 were free of AF (control groups) [13]. Briefly, they sourced data from biobanks of 6 European population-based studies (i.e., The Nord-Trøndelag Health Study (HUNT), deCODE, the Michigan Genomics Initiative (MGI), DiscovEHR, UK Biobank, and the AFGen Consortium) to discover novel signals associated with atrial fibrillation. We used their published list of known European AF-linked SNPs and evaluated each in our cohort to study associations with AF in Puerto Rican Hispanics.

### 2.3. Genetic Data Acquisition from Existing Cohorts of Caribbean Hispanics

The first source of data came from the published study entitled “Pharmacogenetics of Warfarin in Puerto Ricans” (http://clinicaltrial.gov identifier NCT01318057), which was conducted as an observational, open-label, retrospective study of pharmacogenetic associations between candidate genes and effective warfarin dosing in a cohort of Puerto Rican patients [12]. This study was active from January 2008 through July 2010 and recruited Puerto Ricans, mostly older men, between the ages of 21 and 90 years. The participants received stable daily warfarin doses for the treatment and prevention of thromboembolic conditions at an outpatient anticoagulation clinic managed by the Veteran Affairs of the Caribbean Healthcare System (VACHS) in San Juan, PR. A full description of this cohort and detailed information on the patient's recruitment process can be found elsewhere [14]. Individual DNA specimens from participants were then used to perform next-generation sequencing of candidate genes (e.g., *CYP2C9* and *VKORC1*) and various genetic tests with different methods, including a total genome screening with the Infinium™ Human OminiXpress-24 BeadChip array (~650 K markers). The study was supported in part by the National Heart, Lung and Blood Institute (NHLBI, grants # HL123911), the MBRS SCORE Program at the National Institute of General Medical Sciences (NIGMS), and other local funding mechanisms and approved by the Institutional Review Board (IRB) at both VACHS (#00558) and UPR-MSC (A4070109).

A total of 106 participants from this original study of warfarin pharmacogenetics were found to have an indication of warfarin for self-reported atrial fibrillation. Still, only 66 of these patients had the entire genomic data available for further assessments and were included in this secondary analysis as *cases*. None of these patients had active malignancies, structural or valvular heart disease, or liver disease during recruitment.

The second data source came from an ongoing study entitled “A Genomic Approach for Clopidogrel in Caribbean Hispanics” (http://clinicaltrial.gov identifier NCT03419325). This study is a nonrandomized, parallel assignment, open-label interventional clinical trial funded by the National Institute on Minority Health and Health Disparities (NIHMD, U54 grant # MD007600-31) [13]. The IRB also approved the study at UPR-MSC (A4070417). This study started in January 2018 and is an ongoing multicenter clinical trial, conducted at the Cardiovascular Center of Puerto Rico and the Caribbean and the Pavia Hospital in San Juan, PR. It recruits self-reported Puerto Rican Hispanic men or women living in Puerto Rico, older than 21 years, who use clopidogrel with indications for primary or secondary prevention of cardiovascular diseases. All participants underwent a thorough interview to assess past medical history. Afterward, all qualifying participants underwent genetic testing with the Infinium™ Multi-Ethnic AFR/AMR Mega chip array (~1.4 M markers; Illumina, San Diego, CA).

A total of 459 participants from the study of clopidogrel pharmacogenomics were included in the present study's secondary analysis. However, 3 of these participants were identified to have a diagnosis of atrial fibrillation. They were considered as *cases* for this secondary analysis. From this study, 456 patients were added to the *control* group for said analysis. The final sample size was 555 patients (i.e., 69 cases and 455 controls).

### 2.4. Candidate Gene Association Analysis

The candidate gene association analysis was performed using the available genotype dataset of the 111 loci of interest highly suggestive of AF in Europeans. To this purpose, patients were classified as cases and controls based on their AF status. The corresponding association analyses were carried out in program PLINK v.1.07 by using the --*assoc* and --*logistic* model options, at a 5% significance level and following a Bonferroni-adjusted multiple comparison method. log_10_ (*p* values) were plotted against the genomic position using Locus Zoom.

Quality control measures were implemented on samples by assessing for HapMap concordance, Mendelian consistency, reproducibility, and SNP completeness of >99.5%. Samples were checked for annotated sex or genetically determined sex/gender, deviations in heterozygosity, gross chromosomal anomalies, unexpected duplicates or relatedness, missing call rates, contamination, and batch effects or population outliers—portions of the genome with significant chromosomal anomalies where be filtered out. Quality metrics were used to filter SNPs before imputation and association testing: missing call rate (>2%), high Mendelian errors, and pairwise genetic similarity analysis to identify duplicate-sample discordance and deviation from Hardy-Weinberg equilibrium (*p* > 0.05). SNPs were removed from the initial list of autosomal markers based on the following criteria: markers classified as call rate below the threshold, off-target variants, minor allele frequency < 1%, and missing genotyping rate per SNP > 5%.

When necessary, genotype imputation was performed with IMPUTEv2, using the combined 1000 Genomes Project phase 1 reference panel (1000 genomes phase I-integrated haplotypes, NCBI build b37, release date December 2013, no singletons). Samples were imputed together with genotyped SNPs that pass quality filters and represent unique positions on the autosomes and nonpseudo autosomal parts of the X chromosome. Imputations were carried out in two-step directions: SHAPEIT (v.2.r644) was used for prephasing, followed by imputation from the reference panel into the estimated haplotypes with IMPUTE (v.2.3.0) software. Variants with at least two copies of the minor allele and present in any of the four 1000 genomes continental panels were imputed. Quality controls included examining the “info score < 0.6,” masked SNP r2, and the ratio of observed variance of imputed dosages to the expected binomial variance. The results of the association analysis were filtered according to an “effective minor allele count.”

### 2.5. Admixture Analysis

Continental-ancestry proportions were estimated with a model-based analysis using the ADMIXTURE software package [15], under the assumption of three ancestral populations (i.e., African: Yoruba in Ibadan, from Nigeria (YRI), European: European of Iberian descent from Spain (IBS), and Native American (NAT) for *k* = 3) in the admixture model. The NAT reference was taken from (REFERENCE NAT). The other applied parameters were ten independent runs with 70,000 burn-in steps and 30,000 Markov Chain Monte Carlo replicates [16]. To evaluate population stratification and the effect of admixture as a confounder, we used the EIGENSTRAT method implemented in EIGENSOFT software to perform a principal component (PC) analysis of the study sample pruning markers showing LD. Both ancestry proportions and the principal components were used to adjust model predictions [17].

### 2.6. Machine Learning (ML) Algorithms

Genomic data at each locus of interest, from each SNP, detected in the prior GWAS, was analyzed as an additive variant in the ML algorithms. Additional information on relevant clinical and demographic covariates that were expected to contribute to the AF phenotype was either added to the model algorithm or computed using the *lm* function in R (version 2.15.3). We divided the data as 80% for the training set and 20% for the testing set. The training set had an imbalanced distribution for the number of *controls* versus *cases*. A randomized oversampling technique was then used to balance the training dataset to develop the models [18].

Five ML algorithms were trained to generate the models using the training set. The models were validated using the testing set. The five ML algorithms were Random Forest (RF) [19], Logistic Regression (LR) [20], Support-Vector Machine (SVM) [21], Gradient Boost (GB) [22], and AdaBoost (AB) [23]. We used the implementation of these algorithms from the sci-kit learn Python library [24].

### 2.7. Statistical Analysis

We utilized the genomic data of each qualifying individual and assessed the list of SNPs reported. We then compared each participant of the said SNP groups with the published 111 SNP associated with AF in the European biobank genomic analysis [11]. We selected 13 SNPs that presented with the highest receiver operator curve analysis to diagnose atrial fibrillation in the model. We indicated a “2” if the SNP from the European list was homozygous, “1” if it was heterozygous, or a “0” if absent or nullizygous.

Admixture data was reported by the percentage of ancestry per Yoruba, Native American, and European-European of Iberian descents. We implemented a correlational analysis with Pearson Product-Moment Coefficient (*r*^2^) and multiple regression to estimate the association between genetic ancestry fraction and atrial fibrillation. This model was used to show if there is an association between variables that are not purely correlational.

SNP data were transformed into categorical data to evaluate associations between AF and European AF snips' presence. First, we categorized the genetic snip variables by no mutation or nullizygous, heterozygous, and homozygous. We then created three categories based on the amount of total positive snips and created a range from 0 to 26. The following groups were created: less than 5, 5 to 12, and larger or equal to 13, according to Fisher's exact test with a significance level of 0.05.

We categorized admixture according to the most predominant ethnicity (i.e., European, African, and Native American ancestry) and perform an analysis of the association between AF status. We used Fisher's exact test with a significance level of 0.05 to evaluate for the association. Finally, we used a linear regression analysis after categorizing SNP by “no mutation,” “heterozygous,” or “homozygous,” and compared with AF status, reported as an odds ratio.

We performed a machine learning analysis using the following metrics: accuracy, precision, recall, *F*-score, and area under (AUC) the receiver operating curve (ROC). We focused our decision on the best model on the AUC metric results. Python 3.5 [25]. was used in conjunction with the Scikit-Learn v0.20.2 machine learning module [26] to compare the ML algorithms and develop the final model.

## 3. Results and Discussion

In the sample displayed in Table 1, we see 555 Puerto Rican Hispanic patients, with male predominance (61.62%). Within the male population, 49.55% have a negative diagnosis of atrial fibrillation. The median age was 69 years, with an interquartile range of IQR = 15. The general analysis for categorical SNP copies demonstrated statistical significance (*p* value <0.001) between having an AF status, but failed to demonstrate a directionality or distinct pattern.

### 3.1. Admixture Percentage Analysis

**Figure 1:**
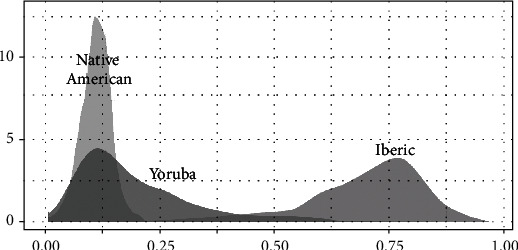
Density plots for the European, Native American, and African ancestry percentage.

In Table 2, we have the means and the standard deviations for each of the ethnicity admixture groups. The presence of European of Iberian descent is more significant than the other groups (mean *I* = 0.69 vs. mean NA = 0.11 vs. mean *Y* = 0.19). Native American admixture displays less variability compared with the European of Iberian descent and African ancestry. We evaluated for atrial fibrillation status and found no difference in the genetic admixture percentage. It is noteworthy that there was no patient with predominant ethnic classification for Native Americans among PRHs. According to Fisher's exact test with a significance level of 0.05, there was no statistical evidence to reject the null hypothesis when evaluating the association between AF and the percentage of genetic admixture.  Figure 1 displays the density for each ethnicity (European of Iberian descent, Native American, and African).

### 3.2. Candidate Single Nucleotide Polymorphisms Associated with Atrial Fibrillation in PRH

Table 3 shows 13 of the 111 genes that were found in our PRH population. The three genes with the highest homozygous and heterozygous frequency were rs7508 (42.79%, 19.70%), followed by rs284277 (47.74%, 17.11%) and rs12426679 (47.74%, 17.117%). We fitted a linear regression model to predict the presence of atrial fibrillation for each gene.

**Figure 2:**
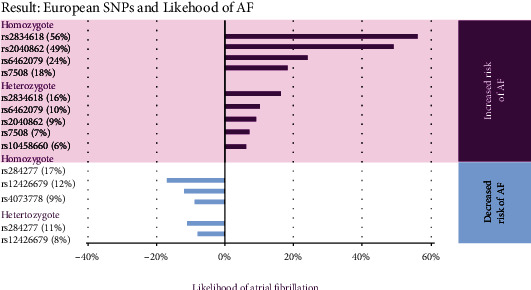
The SNPs with statistical significance organized by the strength of association to AF among PRH.

We found that genes rs2834618, rs2040862, rs6462079, and rs7508 had the strongest association with atrial fibrillation among the PRH cohort. The homozygous rs2834618 had the strongest association with 56% (95% CI: (1.347, 1.803)) times as large as the odds of having AF in nullizygous rs2834618. It was followed by homozygous rs2040862, with 49% (95% CI: (1.258, 1.775)) times as large as the odds of having AF in nullizygous rs2040862.  Figure 2 displays all the significant results organized by the strength of association per category. The homozygous genes rs284277, rs12426679, and rs4073778 were found less likely to be present in patients with AF positive, with odds of not having AF at 17% (95% CI: (0.764, 0.896)), 12% (95% CI: (0.803, 0.962)), and 9% (95% CI: (0.834, 0.982)) times lower compared to the nullizygous group.

### 3.3. Machine Learning Analysis

**Figure 3:**
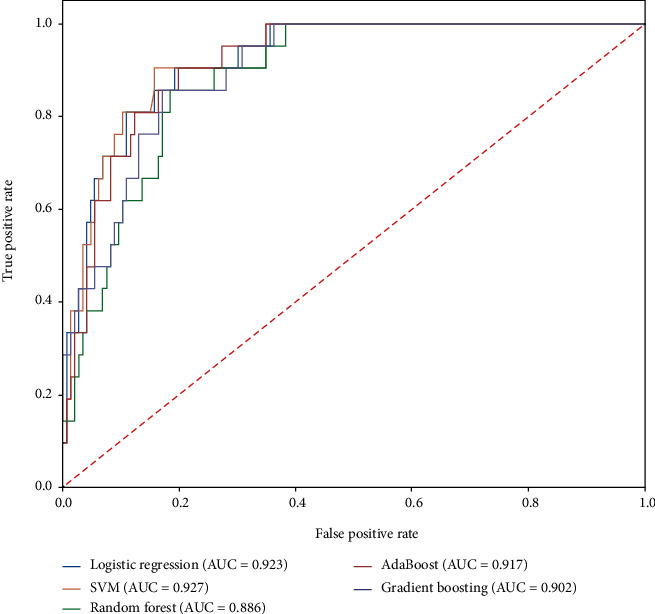
The area under the curve for each fitting model used in the machine learning analysis.

The results of a machine learning analysis shown in Table 4 demonstrated Support Vector Machine (SVM) as the best model to predict AF status (AUC 0.927, Figure 3) based on the variables displayed in Table 5. The strongest predictors for developing AF were the Native American ancestry (3.78), history of heart failure (3.71), rs2834618 (0.76), rs6462079 (0.53), rs7508 (0.53), and type 2 diabetes (0.25). The strongest negative predictors were the use of aspirin (1.58), rs284277 (1.00), rs883079 (0.72), rs10873298 (0.64), rs4073778 (0.58), rs133902 (0.52), rs12426679 (-0.46), and rs2040862 (0.12).

## 4. Discussion

Despite recent advancements in AF's genetic basis, there is much we do not know about its pathogenesis. Extensive genome-wide association studies on AF have shown promising results. However, these studies mostly recruit European descent individuals and systematically underrepresent the Hispanic population. As a result, published SNPs associated with atrial fibrillation have limited generalizability and role among Latino minorities. The Latino population is a challenging cohort mainly because of its inherent genetic heterogeneity, including a rich genetic background (i.e., Cuban, Dominican, Puerto Rican, etc.). This issue requires that we appropriately adjust for confounders. For the first time, we present the relationship between the most robust SNPs associated with AF in a European population and a Puerto Rican Hispanic cohort.

Non-Hispanic whites are presenting with higher AF prevalence than other racial minorities living in the mainland USA [27]. In two hospital-based studies, Puerto Rican Hispanics had a lower prevalence of atrial fibrillation of 2.5% than 5.7% among non-Hispanic Whites. This data is particularly controversial because Hispanics possess a higher prevalence of traditional risk factors for developing AF yet have a lower AF prevalence. This phenomenon is known as the atrial fibrillation paradox. For example, Hispanics and African Americans have higher rates of hypertension, obesity, and dyslipidemia, but have a lower incidence of AF. In particular, Puerto Rican Hispanic women have higher rates of obesity and diabetes mellitus than other Hispanic subgroups. This phenomenon could be explained, in part, by the multiple barriers to healthcare access that plague racial minorities. These barriers may lead to statistical misrepresentation and underreporting of AF status in racial minorities under certain conditions. Nevertheless, further epidemiological studies are required to elucidate this behavior, but it will not be without its challenges.

So far, literature has described traditional risk factors as the best predictors for developing AF. These factors do not always predict AF status. In some cases, patients have cumulative predictors of developing AF, such as a history of heart failure, valvopathies, and enlarged atrial chamber, but never develop AF. Moreover, in other cases, patients develop AF in the absence of traditional risk factors. Familial AF has also been described, further highlighting the genetics role in this condition's pathogenesis.

In 2018 the first genetic study of AF in Latinos was able to identify European-related AF SNPs in a Mexican American cohort. It showed the rs10033454 SNP, chromosome 4q25 (near PITX2), significantly associated with developing AF by as much as a 2.3-fold increase [28]. This SNP was linked to the phenotypical expression of proteins in charge of atrial action potential alterations causing ectopic trigger activity at the pulmonary veins. In our analysis, we did not find rs10033454 to be associated with AF in PRH. We did identify 5 SNP with strong association to AF in a PRH cohort: SNPs: rs2834618, rs6462079, rs7508, rs2040862, and rs10458660.

The first identified SNP was the rs7508. The closest gene to this SNP was the ASAH1. This gene was associated with acid ceramide production for lysosomal activity important for ceramide break down. This rs7508 has been associated with AF based on a study from the Cardiovascular Health Study [29]. Secondly, the rs 2040862, nearest to the WNT8a gene, was associated with AF in our PRH cohort. WNT8a is part of the Wnt signaling pathway, and its expression is linked to increased collagen deposition and fibrosis around atrial cardiomyocytes, which is essential for providing a substrate for AF to develop [30].  Table 6 organizes each relevant SNP and the closest gene of association.

We used a novel machine learning approach and found the Support Vector Machine to be the best discrimination (AUC 0.93) (Table 4 and Figure 3). However, all the predictive models demonstrated proficiency at predicting AF status, with an AUC above 0.88. Overall, Native American admixture demonstrated the strongest association through every model. Other variables such as heart failure, aspirin, rs2834618, rs284277, rs2834618, rs883079, rs10873298, rs4073778, rs6462079, rs7508, and rs133902 demonstrated a coefficient of association above 0.5.

Our study has several limitations. First, we had a small patient sample of which 12% had atrial fibrillation, and women were underrepresented with only two positive cases. Also, more associations can be found as the sample size increases. Second, because there are not many genetic studies focused on PRH, it was deemed necessary to perform a secondary analysis. This type of design could introduce confounders to the results. Third, it would have been optimal to perform a long-term study in which AF status could be followed up to assure proper disease classifications. Also, this study relied on patient-reported AF status. It would have been optimal to have EKG data and echocardiographic data. We did not use Bonferroni correction methods in our analysis, nor were we able to adjust data based on classical AF risk factors.

## 5. Conclusions

We have identified 5 SNPs with a strong association with AF for the first time in a PRH cohort (i.e., SNPs with the strongest association: rs2834618, rs6462079, rs7508, rs2040862, and rs10458660). This study needs to be replicated in a longitudinal study with a larger PRH cohort as AF status could change as the sample age. PRH have an average of 69% European ancestry proportion, but this estimate was found not to be significantly correlated to the AF status. Furthermore, the percentage of admixture as an independent variable (i.e., European of Iberian descent, Native American, and African ancestry proportions) did not show a significant association with the risk of AF in univariate analysis. The Support Vector Machine (SVM) model was the method that best fits the available data for the prediction of AF risk using machine learning analysis (i.e., with an AUC of 0.93289) and also suggested that Native American genetic admixture correlates with the AF status in this multivariate analysis.

Accordingly, our data shows that Native American descent could be a valuable opportunity to research AF risk factors in PRH. In fact, it is unclear whether the postulated association with published AF genes varies across different ethnicities, given the lack of multiethnic genetic research in atrial fibrillation. Further studies on this subject would help facilitate the creation of ethnic-specific preventive strategies.

## Figures and Tables

**Table 1:** Demographical information, admixture analysis, and European-related SNP association with AF in PRH.

Atrial fibrillation	Yes	No	Total
Sex			
Female	2 (0.36%)	211 (38.02%)	213 (38.38%)
Male	67 (12.07%)	275 (49.55)	342 (61.62%)
Total	69 (12.43%)	486 (87.57%)	555
SNP mutation copies			
<5	18 (26.09%)	41 (8.44%)	59 (10.63%)
5-12	43 (62.32%)	434 (89.30%)	477 (85.95%)
>12^∗^	8 (11.59%)	11 (2.26%)	19 (3.42%)
Total	69 (12.43%)	486 (87.57%)	555

^∗^ *p* value <0.001 regarding the association between the number of mutations and AF, as per Fisher's exact test.

**Table 2:** Admixture analysis by the number of European-related SNP in PRH.

Atrial fibrillation	Yes	No
Ancestry	Mean ± SD	Mean ± SD
European	0.690838 ± 0.125362	0.698278 ± 0.130031
African	0.197318 ± 0.128053	0.194678 ± 0.136442
Native American	0.111844 ± 0.0349847	0.107045 ± 0.0416816

^∗^ *p* value <0.001 regarding the association between the percentage of admixture between AF status, as per Fisher's exact test.

**Table 3:** Linear regression analysis of the association between each relevant SNPs and AF in Puerto Rican Hispanics. SNPs were chosen from previous reports that showed a significant association with AF in Europeans.

SNPs	Frequency (*n*, %)			OR atrial fibrillation^∗^			
	0	1	2	1		2	
				OR	95% CI	OR	95% CI
rs2834618	314 (56.577%)	203 (36.577%)	38 (6.847%)	1.16	(1.079, 1.238)	1.56	(1.347, 1.803)
rs2040862	303 (54.595%)	208 (37.477%)	44 (7.928%)	1.09	(1.017, 1.169)	1.49	(1.258, 1.775)
rs6462079	255 (45.946%)	237 (42.703%)	63 (11.351%)	1.10	(1.040, 1.168)	1.24	(1.119, 1.382)
rs7508	211 (38.018%)	237 (42.703%)	107 (19.279%)	1.07	(1.010, 1.134)	1.18	(1.063, 1.307)
rs284277	195 (35.135%)	265 (47.748%)	95 (17.117%)	0.89	(0.842, 0.949)	0.83	(0.764, 0.896)
rs12426679	195 (35.135%)	265 (47.748%)	95 (17.117%)	0.92	(0.864, 0.970)	0.88	(0.803, 0.962)
rs10458660	435 (78.378%)	101 (18.198%)	19 (3.423%)	1.06	(1.003, 1.123)	0.97	(0.852, 1.098)
rs4073778	269 (48.468%)	226 (40.721%)	60 (10.811%)	0.95	(0.893, 1.007)	0.91	(0.834, 0.982)
rs883079	199 (35.856%)	266 (47.928%)	90 (16.216%)	0.95	(0.899, 1.012)	0.95	(0.868, 1.036)
rs10873298	269 (48.468%)	226 (40.721%)	60 (10.811%)	0.95	(0.894, 1.010)	0.95	(0.878, 1.023)
rs7612445	314 (56.577%)	203 (36.577%)	38 (6.847%)	0.96	(0.902, 1.104)	1.05	(0.955, 1.148)
rs1458038	438 (78.919%)	103 (18.559%)	14 (2.523%)	1.02	(0.959, 1.078)	1.10	(0.988, 1.233)
rs133902	322 (58.018%)	192 (34.595%)	41 (7.387%)	0.97	(0.927, 1.047)	0.94	(0.865, 1.017)

**Table 4:** Machine learning analysis for the best predictive model fit.

ML model	AUC score
Logistic regression	0.923
Support vector machine	0.927
Random forest	0.886
AdaBoost	0.917
Gradient boosting	0.902

**Table 5:** Coefficients of the association from the SVM.

Variable	Coefficient	Coefficient absolute value
NAT	3.782655	3.782655
CHF	3.710129	3.710129
Aspirin	-1.58356	1.58356
rs284277	-1.00155	1.00155
rs2834618	0.761455	0.761455
rs883079	-0.71725	0.71725
rs10873298	-0.63843	0.63843
rs4073778	-0.57691	0.57691
rs6462079	0.533001	0.533001
rs7508	0.526028	0.526028
rs133902	-0.52396	0.52396
rs12426679	-0.45646	0.45646
Diabetes	0.245134	0.245134
rs2040862	-0.12302	0.12302
Height	0.099007	0.099007
Age	0.091398	0.091398
rs1458038	0.06731	0.06731
IBS	-0.03918	0.03918
rs7612445	0.013199	0.013199
Weight	0.011875	0.011875

NAT stands for Native American ancestry and IBS stands for European of Iberian descent populations in Spain, which is one of the reference populations from the 1000 Genomes Project used in this study to estimate the proportion of European ancestry. CHF stands for congestive heart failure.

**Table 6:** Published data from the GWAS catalogue of relevant SNPs associated with AF in PRH.

Variant and risk allele	*p* value	RAF	OR	CI	Mapped gene	Reported trait
rs284277-C	1 × 10^−9^	0.383	1.04	(1.03-1.06)	CASZ1	Atrial fibrillation
rs7508-A	6 × 10^−9^	0.73	1.1	(1.06-1.13)	ASAH1	Atrial fibrillation
rs2834618-T	3 × 10^−9^	0.9	1.12	(1.09-1.14)	LINC01426	Atrial fibrillation
rs883079-C	1 × 10^−9^	0.29	—	(0.33-0.65)	TBX5	QRS duration
rs10873298-C	7 × 10^−9^	0.366	1.04	(1.03-1.06)	AC007686.4, LINC01629	Atrial fibrillation
rs4073778-A	5 × 10^−9^	0.564	1.05	(1.04-1.06)	CASQ2	Atrial fibrillation
rs6462079-A	9 × 10^−9^	0.721	1.05	(1.03-1.06)	CREB5	Atrial fibrillation
rs133902-T	9 × 10^−9^	0.427	1.04	(1.03-1.06)	MYO18B	Atrial fibrillation
rs12426679-C	5 × 10^−9^	0.472	1.04	(1.03-1.05)	AC078923.1	Atrial fibrillation
rs2040862-T	3 × 10^−9^	0.18	1.12	(1.07-1.17)		
rs10458660-G	7 × 10^−9^	0.173	1.06	(1.04-1.07)	LRMDA	Atrial fibrillation

## Data Availability

The data that support the findings of this study are openly available in an Institutional OneDrive File at https://1drv.ms/u/s!AreEFHiR_YD0aSaoeLNAtYGs6nM?e=7GZEAP. The data that support the findings of this study are openly available in dbGaP: Genotypes and phenotypes at https://www.ncbi.nlm.nih.gov/projects/gapprev/gap/cgi-bin/preview1.cgi?GAP_phs_code=sLkvEjCcxkGrlRcDdbGaP, Study Accession: phs001496.v1.p1.

## References

[1] Atrial Fibrillation. https://www.mayoclinic.org/diseases-conditions/atrial-fibrillation/symptoms-causes/syc-20350624#:~:text=faster%20than%20normal.-,Atrial%20fibrillation%20is%20an%20irregular%20and%20often%20rapid%20heart%20rate,to%20175%20beats%20a%20minute.

[2] Who is at Risk for Atrial Fibrillation (AF or AFib)?American Heart Association. https://www.heart.org/en/health-topics/atrial-fibrillation/who-is-at-risk-for-atrial-fibrillation-af-or-afib.

[3] Atrial fibrillation - Symptoms and causes - Mayo Clinic. https://www.mayoclinic.org/diseases-conditions/atrial-fibrillation/symptoms-causes/syc-20350624#:~:text=During%20atrial%20fibrillation%2C%20the%20heart's,shortness%20of%20breath%20and%20weakness.

[4] Shen A. Y.-J., Contreras R., Sobnosky S. (2010). Racial/ethnic differences in the prevalence of atrial fibrillation among older adults - a cross-sectional study. *Journal of the National Medical Association*.

[5] Studies to Treat or Prevent Pediatric Type 2 Diabetes (STOPP-T2D) Prevention Study Group (2008). Prevalence of the metabolic syndrome among a racially/ethnically diverse group of U.S. eighth-grade adolescents and associations with fasting insulin and homeostasis model assessment of insulin resistance levels. *Diabetes Care*.

[6] Borzecki A. M., Bridgers D. K., Liebschutz J. M., Kader B., Kazis L. E., Berlowitz D. R. (2008). Racial differences in the prevalence of atrial fibrillation among males. *Journal of the National Medical Association*.

[7] Noel S. E., Arevalo S., Smith C. E. (2017). Genetic admixture and body composition in Puerto Rican adults from the Boston Puerto Rican Osteoporosis Study. *Journal of Bone and Mineral Metabolism*.

[8] Roselli C., Chaffin M. D., Weng L.-C. (2018). Multi-ethnic genome-wide association study for atrial fibrillation. *Nature Genetics*.

[9] Bunney P. E., Zink A. N., Holm A. A., Billington C. J., Kotz C. M. (2017). Orexin activation counteracts decreases in nonexercise activity thermogenesis (NEAT) caused by high-fat diet. *Physiology & Behavior*.

[10] Genetics research is overwhelmingly white, and holding back precision medicine - Vox. http://www.vox.com/science-and-health/2018/10/22/17983568/dna-tests-precision-medicine-genetics-gwas-diversity-all-of-us.

[11] Nielsen J. B., Thorolfsdottir R. B., Fritsche L. G. (2018). Biobank-driven genomic discovery yields new insight into atrial fibrillation biology. *Nature Genetics*.

[12] Valentín I. I., Rivera G., Nieves-Plaza M. (2014). Pharmacogenetic association study of warfarin safety endpoints in Puerto Ricans. *Puerto Rico Health Sciences Journal*.

[13] Duconge J. A genomic approach for clopidogrel in Caribbean Hispanics-full text view-clinical. https://clinicaltrials.gov/ct2/show/NCT03419325.

[14] Duconge J., Ramos A. S., Claudio-Campos K. (2016). A novel admixture-based pharmacogenetic approach to refine warfarin dosing in Caribbean Hispanics. *PLoS One*.

[15] Yuan K., Zhou Y., Ni X., Wang Y., Liu C., Xu S. (2017). Models, methods and tools for ancestry inference and admixture analysis. *Quantitative Biology*.

[16] Chikhi L., Bruford M. W., Beaumont M. A. (2001). Estimation of admixture proportions: a likelihood-based approach using Markov Chain Monte Carlo. *Genetics*.

[17] 1000 Genomes Project Consortium, Auton A., Brooks L. D. (2015). A global reference for human genetic variation. *Nature*.

[18] Witten I. H., Frank E., Hall M. (2011). Machine Learning and Statistics, Data mining: practical machine learning tools and techniques. *Morgan Kaufmann*.

[19] Boehmke B., Greenwell B., Boehmke B., Greenwell B. (2019). Random forests. *Hands-On Machine Learning with R*.

[20] Le Cessie S., Van Houwelingen J. C. (1992). Ridge estimators in logistic regression. *Applied Statistics*.

[21] Huang X., Maier A., Hornegger J., Suykens J. A. K. (2017). Indefinite kernels in least squares support vector machines and principal component analysis. *Applied and Computational Harmonic Analysis*.

[22] Petrere-Jr M. (2014). Greedy function approximation: A gradient boosting machine. *The Annals of Statistics*.

[23] Barmaki R. Multimodal assessment of teaching behavior in immersive rehearsal environment - Teach Liv E™.

[24] Varoquaux G., Buitinck L., Louppe G., Grisel O., Pedregosa F., Mueller A. (2015). Scikit-learn: Machine Learning Without Learning the Machinery. *GetMobile: Mobile Computing and Communications*.

[25] Python Software Foundation|Python Software Foundation. http://www.python.org/psf/.

[26] Pedregosa F., Varoquaux G., Gramfort A. (2011). Scikit-learn: Machine Learning in Python. *Journal of Machine Learning Research*.

[27] Ho M. V., Lee J.-A., KCM (2012). 基因的改变NIH Public Access. *Bone*.

[28] Chalazan B., Mol D., Sridhar A. (2018). Genetic modulation of atrial fibrillation risk in a Hispanic/Latino cohort. *PLoS One*.

[29] Jensen P. N., Fretts A. M., Hoofnagle A. N. (2020). Plasma ceramides and sphingomyelins in relation to atrial fibrillation risk: the cardiovascular health study. *Journal of the American Heart Association*.

[30] Guo F., Yi X., Li M., Fu J., Li S. (2017). Snail1 is positively correlated with atrial fibrosis in patients with atrial fibrillation and rheumatic heart disease. *Experimental and Therapeutic Medicine*.

